# Heat shock factor 1 inhibits the mitochondrial apoptosis pathway by regulating second mitochondria-derived activator of caspase to promote pancreatic tumorigenesis

**DOI:** 10.1186/s13046-017-0537-x

**Published:** 2017-05-08

**Authors:** Wenjin Liang, Yong Liao, Jing Zhang, Qi Huang, Wei Luo, Jidong Yu, Jianhua Gong, Yi Zhou, Xuan Li, Bo Tang, Songqing He, Jinghong Yang

**Affiliations:** Department of Hepatobiliary Surgery, Guilin Medical University, Affiliated Hospital, Guilin, 541004 Guangxi People’s Republic of China

**Keywords:** Pancreatic cancer, Heat shock factor 1, Heat shock proteins, Oncogene, SMAC, Apoptosis, Proliferation

## Abstract

**Background:**

As a relatively conservative transcriptional regulator in biological evolution, heat shock factor 1 (HSF1) is activated by, and regulates the expression of heat shock proteins (HSPs) in response to a variety of stress conditions. HSF1 also plays a key role in regulating the development of various tumors; however, its role in pancreatic cancer and the specific underlying mechanism are not clear.

**Methods:**

We first examined HSF1 expression in pancreatic cancer tissues by immunohistochemistry, and then studied its clinical significance. We then constructed HSF1-siRNA to investigate the potential of HSF1 to regulate apoptosis, proliferation and the cell cycle of pancreatic cancer cells and the underlying mechanism both in vitro and in vivo. Protein chip analysis was used subsequently to explore the molecular regulation pathway. Finally, second mitochondria-derived activator of caspase (SMAC)-siRNA was used to validate the signaling pathway.

**Results:**

HSF1 was highly expressed in pancreatic cancer tissues and the level of upregulation was found to be closely related to the degree of pancreatic cancer differentiation and poor prognosis. After HSF1-silencing, we found that pancreatic cancer cell proliferation decreased both in vitro and in vivo and the apoptotic cell ratio increased, while the mitochondrial membrane potential decreased, and the cells were arrested at the G0/G1 phase. In terms of the molecular mechanism, we confirmed that HSF1 regulated SMAC to inhibit mitochondrial apoptosis in pancreatic cancer cells, and to promote the occurrence of pancreatic tumors. SMAC silencing reversed the effects of HSF1 silencing.

**Conclusion:**

Our study provides evidence that HSF1 functions as a novel oncogene in pancreatic tumors and is implicated as a target for the diagnosis and treatment of pancreatic cancer.

**Electronic supplementary material:**

The online version of this article (doi:10.1186/s13046-017-0537-x) contains supplementary material, which is available to authorized users.

## Background

Pancreatic cancer is a malignant tumor of the digestive tract associated with high mortality. Furthermore, early diagnosis is difficult and the cancer is commonly associated with drug resistance [1]. The National Center for Health Statistics of the United States of America reported in 2015 that the new incidence and mortality of pancreatic cancer accounted for 3 and 7%, respectively, of all tumors [2]. The Chinese Center for Cancer Control and Prevention reported that the incidence and mortality of pancreatic cancer accounted for 2.1 and 2.8%, respectively, of all tumors in the period between 2009 and 2011 [3]. Despite significant progress in the diagnosis and treatment of pancreatic cancer in recent years, surgery remains the only effective approach to treatment of the tumor and improvement in the prognosis of patients. Moreover, to date, there have been no obvious improvements in the postoperative five-year survival and recurrence rates. Therefore, the molecular characteristics of patients with advanced or refractory cancer are being explored actively to identify new tumor markers that can be used to guide clinical treatment strategies [4].

Heat shock factors (HSFs) are important protective regulators of responses to various kinds of acute stress. These factors function as inducible transcriptional regulators of molecular chaperones and other stress proteins [5]. The HSF family comprises four members, which participate in the normal growth of organisms and increase the lifespan of a variety of regulatory signaling pathways. HSF1 is one of the most important members of the HSF family [6] and is activated in response to stress conditions such as heat stimulation, infection and toxicity. Following activation, HSF1 forms tripolymers, which regulate the translation of various HSPs, such as HSP90, HSP60, and HSP27 [7, 8]. HSPs regulate normal protein folding by preventing protein mismatches and the formation of polymers and also play a key regulatory role in tumor development [8]. HSF1 regulates the activation of PKC and its downstream signaling pathway to inhibit tumor cell apoptosis [9]. HSF1 gene deletion in mice prevents tumorigenesis induced by the ras oncogene or tumor suppressor p53 hotspot mutation. Furthermore, human tumor cells from different sources have been shown to be more dependent on HSF1 than normal cells in maintaining cell growth and proliferation [10]. HSF1 also promotes the development of pancreatic cancer drug resistance [11]. Therefore, as a tumor regulator, HSF1 plays a key regulatory role in inhibiting tumor cell apoptosis and promoting tumor development. It has been reported that HSF1 is highly expressed in pancreatic cancer tissues and inhibits pancreatic cancer cell apoptosis [12, 13]. However, the role of HSF1 in the tumorigenesis of human pancreatic cancer is not yet clear.

In this study, we investigated the ability of HSF1 to regulate the growth and proliferation of pancreatic cancer cells and the underlying mechanism. We confirmed that the expression of HSF1 in pancreatic cancer tissue was significantly higher than that in para-cancerous tissues and was significantly associated with poor clinical prognosis in patients with pancreatic cancer. Following HSF1 silencing, the proliferation of pancreatic cancer cells in vitro and in vivo decreased, the ratio of apoptotic cells increased, the mitochondrial membrane potential decreased, and the cell cycle progression was arrested in the G0/G1 phase. Regarding the molecular mechanism, HSF1 downregulates SMAC expression to inhibit mitochondrial apoptosis in pancreatic cancer cells, thus promoting pancreatic cancer. Thus, our results demonstrate a novel molecular mechanism by which HSF1 promotes pancreatic carcinogenesis and implicate HSF1 as an independent predictor of survival and prognosis in patients with pancreatic cancer. Furthermore, these findings indicate the potential of HSF1 as a novel target for the diagnosis and treatment of pancreatic cancer.

## Methods

### Experimental reagents and antibodies

Liposome and total RNA extraction reagents (TRIzol) were purchased from Invitrogen (Grand Island, NY, USA). HSF1, Ki-67, caspase 3, caspase 8, Bax, Bcl-2, SMAC and XIAP antibodies were purchased from Santa Cruz Biotechnology (Dallas, TX, USA). CyclinD1, CDK6 and β-actin antibodies were purchased from Origene (Rockville, MD, USA). Other reagents were purchased from Sigma (St. Louis, MO, USA). Small interfering RNA (siRNA) was purchased from GenePharma (Shanghai, China). Transfected liposomes were purchased from Invitrogen.

### Patient data and clinical specimens

A total of 50 pairs of paraffin-embedded pancreatic cancer and para-cancerous tissue specimens and corresponding clinical data were collected at random from the pancreatic cancer patients who received hepatobiliary and pancreatic surgical resection and were confirmed by pathologists at the Affiliated Hospital of Guilin Medical University (Guangxi, China) from 2007 to 2014. TNM staging was performed according to the TNM staging system revised in 2002 by the International Union against Cancer (UICC) for clinicopathological parameters such as sex, age, smoking history, tumor location, size and diameter, histological type and staging. Fifty patients were followed-up until September 2016. The research protocol was approved by the Ethics Committee of Guilin Medical University and an informed consent form prepared in accordance with the Declaration of Helsinki was signed by the patients.

### Immunohistochemical analysis

The resected pancreatic cancer and para-cancerous tissues were fixed overnight with 4% paraformaldehyde and paraffin-embedded sections (thickness, 4 μm) were prepared. Sections were then processed for immunohistochemical staining according to the EnVision two-step procedure [14]. The results were divided into four grades according to the degree of positive immunohistochemical staining: 0: <10%; 1+: 11%–25%; 2+: 26%–50%; 3+: >50%. Immunohistochemical analysis and scoring were performed by two independent investigators.

### Cell culture and transfection

Four pancreatic cancer cell lines (PANC-1, BXPC-3, ASPC-1 and CFPAC-1) were purchased from the Cell Bank of the Chinese Academy of Sciences (Shanghai, China). PANC-1 and BXPC-3 were cultured in Dulbecco’s modified Eagle medium (Thermo Fisher Scientific, South America) containing 10% fetal bovine serum (Thermo Fisher Scientific). ASPC-1 was cultured in RPMI-1640 (Thermo Fisher Scientific) containing 10% fetal bovine serum (Thermo Fisher Scientific). CFPAC-1 was cultured in Dulbecco’s modified Eagle medium (Thermo Fisher Scientific) containing 10% fetal bovine serum (Thermo Fisher Scientific). All cell lines were cultured at 37 °C under 5% CO_2_/95% air and revived every 3 to 4 months. HSF1 and second mitochondria-derived activator of caspase (SMAC)-specific siRNAs were prepared and used in transfections as described previously [15].

### Cell proliferation assay

Cells were seeded in a 96-well plate (100 μl/well) at a cell density of 3 × 10^3^ cells/well. After incubation for a certain time, 10 μl of cell counting kit-8 (CCK-8) solution was added and cells were cultured at 37 °C under 5% CO_2_/95% air. After 1–4 h, the absorbance at 450 nm was measured using a microplate reader (Molecular Devices, Thermo Fisher Scientific).

### Colony formation assay

Cells were seeded at 500 cells/well in six-well plates. After two weeks in culture at 37 °C under 5% CO_2_/95% air, the cells were fixed with 4% paraformaldehyde for 20 min and then stained with 1% Crystal Violet (G1062, Solarbio, Japan) overnight. After washing three times with PBS, the number of cells per well was counted.

### Detection of apoptosis

Cells were collected and washed twice with ice-cold PBS before adding 100 μl of Annexin V and propidium iodide (PI; Sigma, USA). Cells were incubated for 20 min at room temperature in the dark prior to flow cytometric analysis (Merck Millipore, Germany).

### Cell cycle analysis

Cells were collected and washed twice with ice-cold PBS before resuspension in 10 ml of 70% ethanol for fixation overnight at −20 °C. Subsequently, the cells were washed twice with PBS before adding RNase Diluent (500 μl at 100 μg/ml; Sigma). Cells were then incubated in a water bath at room temperature for 30 min and stained with PI (25 μl at 1 mg/ml; Sigma, USA) for 5 min in the dark prior to flow cytometric analysis (Merck Millipore).

### Detection of mitochondrial membrane potential

Cells were collected and resuspended in 1 ml of PBS before adding rhodamine 123 (1 μmol/L). Cells were then incubated at 37 °C for 45 min in the dark before observation of fluorescence intensity under a confocal laser scanning microscope (Zeiss, Germany).

### Establishment of HSF1 stable knockdown cell lines

Retroviral constructs containing human pSuper.retro.puro with shRNA against human HSF1 was prepared as described previously [16]. The generation of retrovirus supernatants and transfection of pancreatic carcinoma cells were conducted as described previously [16]. qRT-PCR and Western blotting analysis were used to confirm the expression of HSF1.

### Nude mouse tumor model

Forty male nude mice (aged 5–6 weeks) were purchased from the Animal Experimental Center of Guilin Medical University. The mice were randomly divided into two groups (*n* = 10 per group): the control group (empty plasmid transfected cells) and the experimental group (shHSF1 stably transfected cells). The empty plasmid transfected cells and shHSF1 stably transfected cells were collected in the logarithmic growth phase and washed with PBS to prepare cell suspensions. Cell suspensions (2 × 10^6^ cells) were injected subcutaneously into the right inguinal area. Tumor growth was observed at 7, 14, 21 and 28 days after tumor implantation. After 28 days, nude mice were sacrificed by cervical dislocation, the tumors were excised and the tumor weight was recorded.

All animal experiments were approved by the Animal Care and Use Committee of Guilin Medical University.

### Western blot analysis

Lysis buffer was added to the collected cells and total proteins were extracted. Protein concentration was determined using the BCA (bicinchoninic acid) method [17]. Protein lysates (30 μg) were separated by sodium dodecyl sulfate polyacrylamide gel electrophoresis (SDS-PAGE) and detected using a specific antibody and a peroxidase-labeled secondary antibody. Immunoreactive proteins were visualized by chemiluminescence.

### RT-PCR

Following the addition of total RNA extraction reagent (Grand Island, NY, USA), total RNA was extracted from the collected cells using RNAiso™ Plus kits (Takara, Japan) according to the manufacturer’s instructions. RNA concentration was determined using a spectrophotometer (Beckman Coulter, USA). Reverse transcription was performed using the FastQuant cDNA first strand synthesis kit (TIANGEN, China) and amplified with HSF1-specific primers (Invitrogen, USA) using the TaKaRa RNA PCR kit (AMV, Ver. 3.0) according to the manufacturer’s instructions. The amplified products were analyzed by 1.5% agarose gel electrophoresis.

### Protein chip analysis

The protein samples from different treatment groups were added to the human apoptosis-related semi-quantitative protein chip (Cat# AAH-APO-G1-8, RayBiotech, Norcross, GA, USA) for antigen-antibody reactions, and eluted to remove unreacted proteins. The results were analyzed by fluorescence scanning. Cluster analysis was performed with Cluster v3.0 software, and the specific signaling pathways were identified by gene ontology and KEGG enrichment analysis.

### Statistical analysis

All experimental results were analyzed with SPSS18.0 statistical software. The correlations between the expression of HSF1 and clinicopathological parameters were evaluated by chi-square test (*χ* ^2^), and the quantitative data were evaluated by paired *t*-test. Patient survival rates and prognosis were estimated using the Kaplan–Meier method, and survival curves were compared between using log-rank tests. Data were presented at the mean ± standard deviation (SD) of three independent experiments. *P* < 0.05 was considered to indicate statistical significance.

## Results

### HSF1 was highly expressed in pancreatic cancer tissues and correlated positively with clinicopathological features

**Fig. 1:**
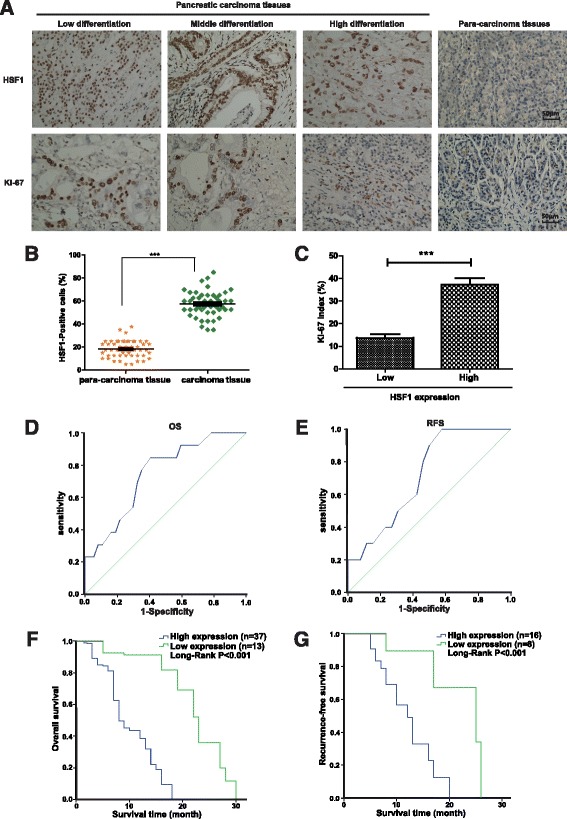
High expression of HSF1 in pancreatic cancer tissues and association with poor prognosis in patients. **a** Representative image of immunohistochemical detection of the expression of HSF1 and Ki-67 proteins in 50 pairs of pancreatic cancer and para-carcinoma tissue specimens. Scale bar, 50 μm. **b** Semi-quantitative analysis of HSF1 protein expression in pancreatic cancer and para-carcinoma tissues. Differences were analyzed by paired *t*-test and data represent the mean ± standard deviation of three independent experiments. ***P < 0.001. **c** Correlation between HSF1 and the proliferation marker, Ki-67, in pancreatic cancer tissues by immunohistochemistry. Figure shows the Ki-67 index in pancreatic cancer tissues with low or high expression of HSF1. **d** and **e** Receiver operating characteristic (ROC) curve analysis of the correlation between the expression of HSF1 protein in patients with pancreatic cancer and overall survival (**d**) and recurrence-free survival (**e**). **f** and **g** Kaplan–Meier survival curve analysis of the correlation between the expression of HSF1 protein in patients with pancreatic cancer and overall survival (**f**) and recurrence-free survival (**g**)

A total of 50 pairs of paraffin-embedded pancreatic cancer and para-cancerous tissue specimens were collected at random for immunohistochemical analysis (Fig. 1a). The number of HSF1-positive cells was significantly higher in the pancreatic cancer tissue specimens compared with that in corresponding the para-cancerous tissue specimens (Fig. 1b). HSF1 expression levels in pancreatic carcinoma were classified as high expression (*n* = 37) and low expression (*n* = 13) according to the method described in the Materials and Methods section. Statistical analysis revealed a significant correlation between HSF1 levels and the degree of pancreatic cancer differentiation (*P* = 0.012); however, there was no significant correlation with the other clinical and pathological features, such as age, sex, smoking history and tumor size, tumor diameter (Table 1). The frequency of Ki-67-positive cells was higher in the HSF1-positive cases than in the HSF1-negative cases. Receiver operating characteristic (ROC) curve analysis showed that HSF1 expression was statistically significant for the determination on the overall survival (OS) (area under the curve (AUC) = 0.744, 95% confidence intervals (CI) 0.596–0.892, *P* = 0.009) and recurrence-free survival (RFS) (AUC = 0.715, 95% CI 0.544–0.886, *P* = 0.048) of the patients (Fig. 1c and d). We further analyzed the correlation between HSF1 expression and clinical prognosis of pancreatic cancer patients by generating Kaplan–Meier survival curves. The median OS time of the patients in the HSF1 high expression group was significantly shorter than that in the HSF1 low expression group (median, 8 months vs. 23 months; *P* < 0.001) (Fig. 1e). The RFS time in the HSF1 high expression group was also significantly shorter than that in the HSF1 low expression group (median, 12 months vs. 25 months; *P* < 0.001) (Fig. 1f). Multivariate Cox regression analysis indicated that high HSF1 expression (relative risk = 0.387; *P* = 0.028) is an independent predictor of OS in patients with pancreatic cancer (Table 2). These results strongly suggested that high HSF1 expression in pancreatic cancer is closely related to the degree of tumor differentiation and poor prognosis in the patients with pancreatic cancer.

**Table 1:** Correlations between HSF1 staining and clinicopathologic characteristics of 50 pancreatic carcinoma patients

Variables	HSF1 staining		Total	*P* value
	Low	High		
Age (y)				
< 45	2 (33%)	4 (67%)	6	0.662
≥ 45	11 (25%)	33 (75%)	44	
Sex				
Male	9 (33%)	18 (67%)	27	0.200
Female	4 (17%)	19 (83%)	23	
Drinking history				
No	6 (21%)	23 (78%)	29	0.314
Yes	7 (33%)	14 (67%)	21	
Tumor location				
Head	7 (27%)	19 (73%)	26	0.877
Body	6 (25%)	18 (75%)	24	
Tumor number				
Solitary	9 (26%)	26 (74%)	35	0.944
Multiple	4 (27%)	11 (73%)	15	
Tumor diameter (cm)				
< 2	8 (24%)	26 (76%)	34	0.562
≥ 2	5 (31%)	11 (69%)	16	
Tumor differentiation				
I-II	9 (45%)	11 (55%)	20	0.012^a^
III-IV	4 (13%)	26 (87%)	30	

Abbreviation: *HSF1* heat shock factor 1

^a^Significant p value

**Table 2:** Multivariate analysis with a Cox proportional hazards regresssion model

Variable	Univariate analysis			Multivariate analysis		
	RR	95% CI	P	RR	95% CI	P
Age <45 years	1.023	0.422–2.480	0.960	1.300	0.479–3.523	0.608
(VS. > 45 years)	1.016	0.553–1.867	0.958	0.939	0.410–2.148	0.881
Gender male (VS. female)	0.726	0.384–1.373	0.324	0.886	0.361–2.177	0.792
Drinking history No (VS. Yes)	1.144	0.616–2.124	0.670	2.232	1.007–4.948	0.048
Tumor location Head (VS. Body)	0.699	0.364–1.343	0.283	0.407	0.153–1.082	0.072
Tumor number Solitary (VS. Multipl)	1.378	0.716–2.650	0.337	0.834	0.340–2.051	0.693
Tumor diameter <2 cm (VS. >2 cm)	0.568	0.287–1.124	0.104	0.567	0.229–1.404	0.220
Tumor differentiation I-II (VS. III-IV)	0.379	0.183–0.784	0.009^a^	0.379	0.146–0.982	0.048^a^

Abbreviation: *RR* relative risk, *CI* confidence interval, *HSF1* heat shock factor 1

^a^Significant *p* value

### HSF1 promoted pancreatic cancer cell proliferation in vitro and in vivo

**Fig. 2:**
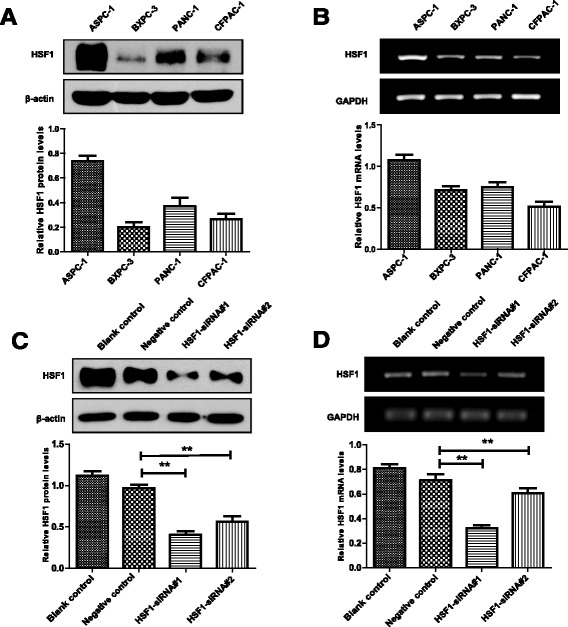
Detection of HSF1 expression in different pancreatic cancer cell lines and HSF1-siRNA screening verification. **a** and **b** Western blot (**a**) and RT-PCR (**b**) were used to detect the expression levels of HSF1 in four pancreatic cancer cell lines (ASPC-1, BXPC -3, CFPAC-1 and PANC-1). **c** and **d** Detection of HSF1 expression in PANC-1 cells after transfection with HSF1-siRNA#1 and HSF1-siRNA#2 by Western blot (**c**) and RT-PCR (**d**). Differences were analyzed by paired *t*-test and data represent the mean ± standard deviation of three independent experiments. *P < 0.05, **P < 0.01, ***P < 0.001

**Fig. 3:**
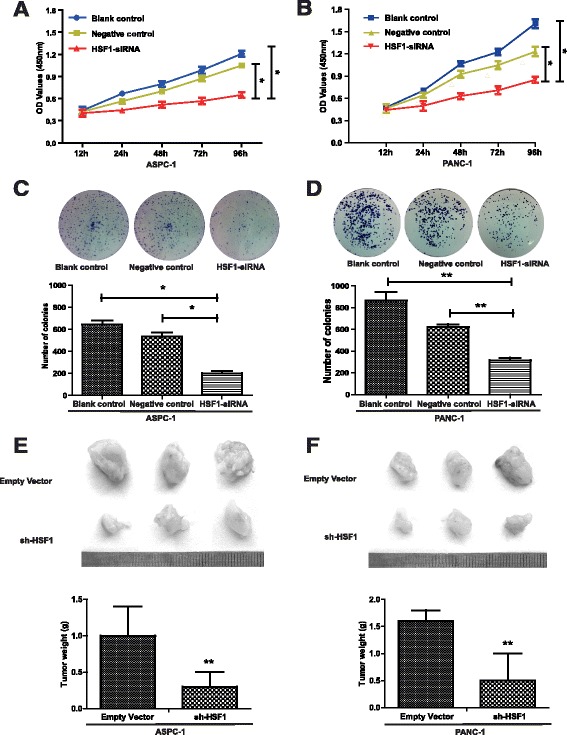
HSF1 promotes pancreatic cancer cell proliferation in vitro and in vivo. **a** and **b** The proliferation of ASPC-1, PANC-1 and their control cells was examined by CCK-8 assay. **c** and **d** Proliferation of ASPC-1, PANC-1 and their control cells was detected by colony formation assay. **e** Representative images of subcutaneous tumors in nude mice (*n* = 3 per group; total = 12 tumors). **f** Tumor weights. Differences were analyzed by paired *t*-test and data represent the mean ± standard deviation of three independent experiments. *P < 0.05, **P < 0.01

We selected four pancreatic cancer cell lines (ASPC-1, BXPC-3, CFPAC-1 and PANC-1) and verified differences in HSF1 expression by Western blot and RT-PCR analyses. The protein and mRNA expression levels of ASPC-1 and PANC-1 cells expressed significantly higher levels of HSF1 mRNA and protein compared with those expressed by CFPAC-1 and BXPC-3 cells (*P* < 0.05) (Fig. 2a and b). We identified optimal siRNA sequences for HSF1 silencing by Western blot and RT-PCR analyses of transfected cells (Fig. 2c and d). After siRNA-mediated silencing of HSF1 in ASPC-1 and PANC-1 cells, tumor cell proliferation was evaluated in CCK-8 assays. SiRNA-mediated HSF1 silencing significantly reduced ASPC-1 and PANC-1 cell proliferation (Fig. 3a, b and Additional file 1: Figure S1A) and colony formation (Fig. 3c, d and Additional file 1: Figure S1B) compared with the blank and negative control groups. Subsequently, we injected nude mice subcutaneously with cells ASPC-1-shHSF1 cells, PANC-1-shHSF1 cells and corresponding cells transfected with the empty vector as a control. In vivo tumor growth was significantly lower in the HSF1 group compared with that in the empty vector group (Fig. 3e and f), suggesting that HSF1 promotes pancreatic cancer cell proliferation both in vitro and in vivo.

### HSF1 regulates apoptosis and cell cycle progression in pancreatic cancer cells

**Fig. 4:**
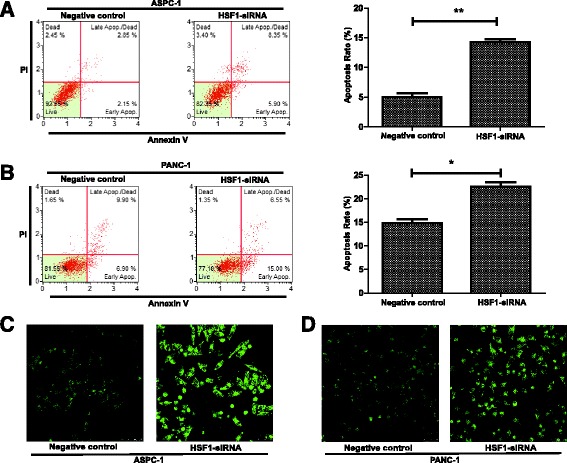
HSF1 regulates the apoptosis of pancreatic cancer cells. **a** and **b** Annexin V/PI double staining showing apoptosis of ASPC-1, PANC-1 and their control cells. **c** and **d** Rhodamine 123 staining showing changes in mitochondrial membrane potential. Differences were analyzed by paired *t*-test and data represent the mean ± standard deviation of three independent experiments. *P < 0.05, **P < 0.01

**Fig. 5:**
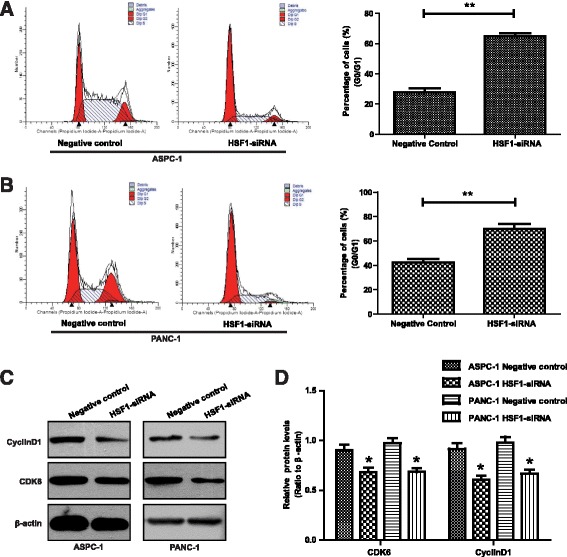
HSF1 regulates the cell cycle in pancreatic cancer cells. **a** and **b** PI staining analysis of the cell cycle of ASPC-1, PANC-1 and their control cells. **c** and **d** Western blot analysis of the expression levels of cyclin D1 and CDK6 proteins. Differences were analyzed by paired t-test and data represent the mean ± standard deviation of three independent experiments. *P < 0.05, **P < 0.01

Although our preliminary results showed that HSF1 promoted pancreatic cancer cell proliferation, the impact of HSF1 on pancreatic cell apoptosis and cell cycle progression required further investigation. Following siRNA-mediated HSF1 silencing, ASPC-1 and PANC-1 cell apoptosis was analyzed by annexin V/PI double staining. Compared with the negative control group, ASPC-1 and PANC-1 cell apoptosis was significantly increased after HSF1 silencing (Fig. 4a, b and Additional file 1: Figure S1C). Furthermore, changes in the mitochondrial membrane potential of ASPC-1 and PANC-1 cells after HSF1 silencing were measured by rhodamine 123 staining. The fluorescence intensity of rhodamine 123 in ASPC-1 and PANC-1 cells increased significantly after HSF1 silencing (Fig. 4c and d), suggesting that mitochondrial membrane potential was significantly decreased by HSF1-silencing. Subsequently, we examined the effects of HSF1 silencing on ASPC-1 and PANC-1 cell cycle progression by PI staining. Compared with the negative control group, the cell cycles of both ASPC-1 and PANC-1 cells were blocked at G0/G1 phase after HSF1 silencing (Fig. 5a, b and Additional file 1: Figure S1D). Western blot analysis also showed that the expression of Cyclin D1 and CDK6 proteins decreased significantly after silencing HSF1 (Fig. 5c, d and e). These results indicated that HSF1 inhibits pancreatic cancer cell apoptosis and regulates the cell cycle to promote tumor cell proliferation.

### HSF1 regulates the expression of SMAC and mitochondrial apoptosis-related proteins

**Fig. 6:**
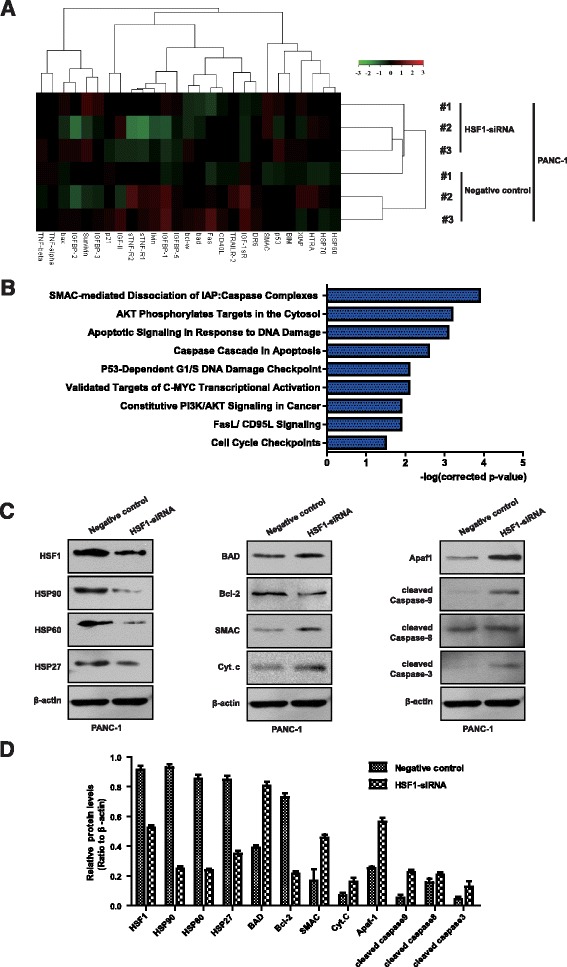
HSF1 regulates the expression of SMAC and mitochondrial apoptosis pathway-related proteins. **a** Protein chip cluster analysis of the expression of apoptosis-related proteins in PANC-1 cells after HSF1 silencing. **b** Protein chip enrichment analysis of the signaling pathway of differentially expressed proteins involved. **c** and **d** Western blot analysis of the expression of HSP90, HSP60, HSP27, Bcl-2, BAD, SMAC, cytochrome C. Apaf1, cleaved caspase 3, cleaved caspase 8 and cleaved caspase 9 proteins in PANC-1 cells after HSF1 silencing. The analysis was repeated three times

To further explore the specific molecular mechanism by which HSF1 promotes proliferation and inhibits apoptosis in pancreatic cancer cells, we compared the expression of apoptosis-related proteins in PANC-1 cells transfected with HSF1-specific siRNA or the empty vector by protein chip cluster analysis. A series of apoptosis-related proteins were significantly differentially expressed following HSF1 silencing (Fig. 6a). We further investigated the signaling pathways associated with these differentially expressed proteins by protein chip enrichment analysis. The results revealed the involvement of these factors in a variety of apoptosis-related signaling pathways, including the phosphorylated AKT-regulated apoptotic pathway, the DNA damage-related apoptosis pathway, the caspase-mediated apoptosis pathway, and the SMAC-mediated mitochondrial apoptosis pathway, which was the most significantly enriched pathway (Fig. 6b). These results suggested that HSF1 regulates the expression of SMAC and mitochondrial apoptosis-related proteins. Therefore, we used Western blot to further verify the expression of mitochondrial apoptotic pathway-related proteins in pancreatic cancer cells before and after silencing HSF1. The expression of HSP90, HSP60, HSP27 and Bcl-2 proteins in PANC-1 cells decreased after HSF1 silencing, while the expression of BAD, SMAC, cytochrome C, Apaf1, cleaved caspase 3 and cleaved caspase 3 were upregulated (Fig. 6c). We also detected the expression of SMAC in 50 pairs of paraffin-embedded sections of pancreatic cancer and para-cancerous tissue specimens using immunohistochemical analysis (Additional file 2: Figure S2A). The frequency of SMAC-positive cells in pancreatic cancer tissue specimens was significantly lower than that in the corresponding para-cancerous specimens (Additional file 2: Figure S2b). Furthermore, SMAC expression correlated negatively with HSF1 expression (Additional file 2: Figure S2c). These results indicated that HSF1 induces HSPs to regulate the expression of SMAC and mitochondrial apoptosis-related proteins.

### HSF1 regulates mitochondrial apoptosis in pancreatic cancer cells via SMAC

**Fig. 7:**
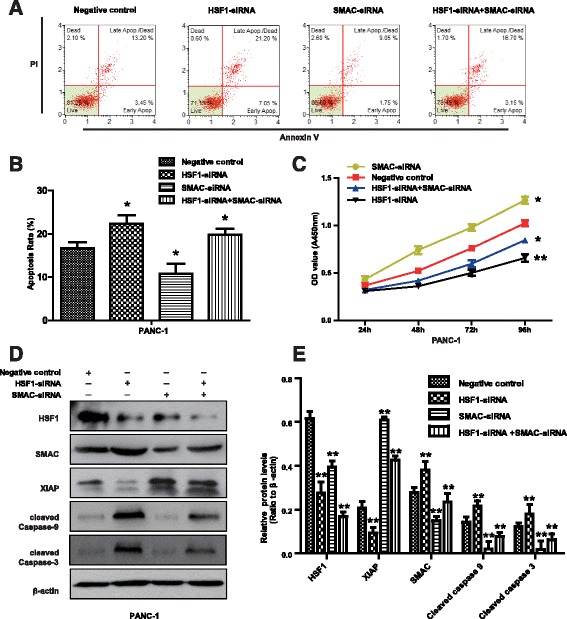
SMAC is a mediator of mitochondrial apoptosis in HSF1-regulated pancreatic cancer cells. **a** and **b** Annexin V/PI double staining was used to detect PANC-1 cell apoptosis. **c** CCK-8 assay of PANC-1 cell proliferation. **d** and **e** Western blot analysis of the expression of SMAC, XIAP, cleaved caspase 3 and cleaved caspase 9 proteins in PANC-1 cells. Differences were analyzed by paired t-test and data represent the mean ± standard deviation of three independent experiments. *P < 0.05, **P < 0.01

**Fig. 8:**
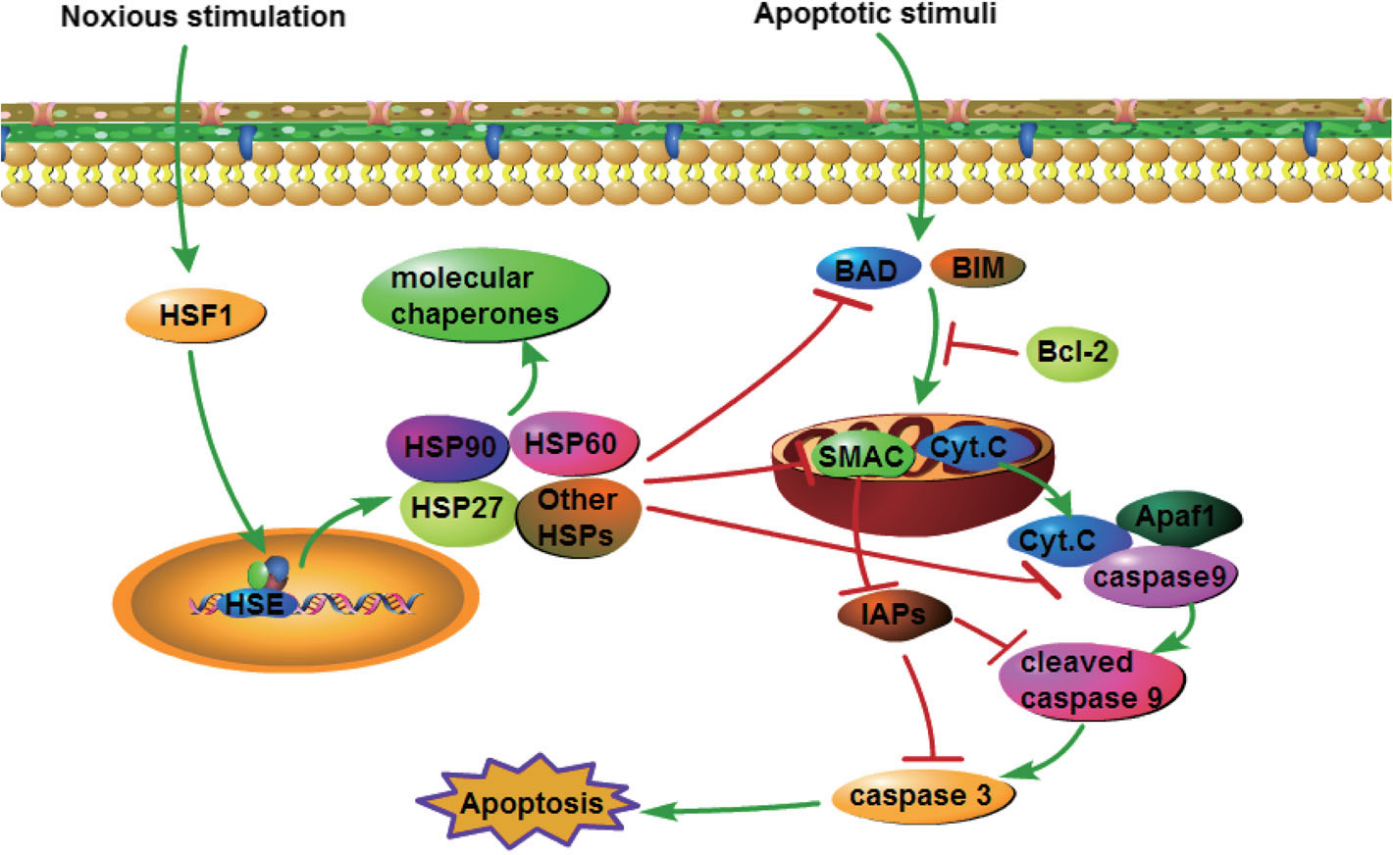
Schematic diagram of the mechanism by which HSF1 regulates pancreatic cancer cell apoptosis

Having shown that HSF1 regulates the expression of SMAC and mitochondrial apoptosis-related proteins, we used siRNA silencing technology to confirm the role of SMAC in the mechanism by which HSF1 regulates mitochondrial apoptosis in pancreatic cancer cells. Compared with the negative control group and HSF1 silenced group, the level of PANC-1 cell apoptosis was significantly decreased by SMAC silencing, while the proliferative capacity was significantly enhanced. However, these effects were ameliorated by co-transfection with HSF1-siRNA and SMAC-siRNA (Fig. 7a, b and c). We then detected the expression of apoptosis-related proteins downstream of SMAC by Western blot analysis. Compared with the negative control group and the HSF1 silenced group, XIAP protein expression was upregulated after SMAC silencing, while the expression levels of cleaved caspase 3 and cleaved caspase 9 proteins were downregulated. However, these effects were ameliorated by co-transfection with HSF1-siRNA and SMAC-siRNA (Fig. 7d and e). These results suggested that SMAC silencing reverses the effects of HSF1 silencing and that SMAC is an intermediate mediator of HSF1 regulation of mitochondrial apoptosis in pancreatic cancer cells. Thus, our results demonstrated that HSF1 inhibits mitochondrial apoptosis in pancreatic cancer cells by downregulating SMAC to promote pancreatic tumorigenesis (Fig. 8).

## Discussion

Pancreatic cancer is a highly malignant digestive system tumor that represents a serious threat to human life because of the difficulties associated with early diagnosis, rapid tumor progression and the high mortality rate [1]. Identification of the key regulatory factors involved in pancreatic tumorigenesis have become a focus of research that is important in the guiding clinical diagnosis and treatment [1]. Many studies have shown that HSF1 is highly expressed and closely related to tumorigenesis and progression in malignancies such as human primary liver cancer [18], breast cancer [19], and melanoma [20]. In the present study, we confirmed that HSF1 is highly expressed in pancreatic cancer tissues compared with normal tissues, and is positively correlated with tumor differentiation and poor prognosis in pancreatic cancer patients. Furthermore, we found that HSF1 expression is positively correlated with the expression of the proliferation marker Ki-67. Moreover, HSF1 is implicated as an independent predictor of survival and prognosis in patients with pancreatic cancer. In terms of the molecular mechanism, we showed that high expression of HSF1 promotes pancreatic cancer cell proliferation both in vitro and in vivo, increases the mitochondrial membrane potential, inhibits apoptosis and regulates the cell cycle. All these effects are reversed by HSF1 silencing.

HSF1 regulates the inhibition of doxorubicin-induced cardiomyocyte mediated by the insulin-like growth factor receptor II (IGF-IIR) signaling pathway [21]. In addition, HSF1 regulates HSPs to maintain the protein balance, thereby inhibiting apoptosis of multiple myeloma cells [22]. HSF1 also regulates the classical Wnt-β-catenin signaling pathway in the inhibition of acute lymphoma cell apoptosis [23]. In the present study, protein chip analysis revealed that SMAC-mediated mitochondrial apoptosis-related proteins were the most significantly enriched in pancreatic cancer cell apoptosis. This pathway is critically involved in responses to a wide range of stresses, including growth factor deficiency, DNA damage, endoplasmic reticulum and death receptor expression. Stress signals result in activation of the Bcl-2 family of apoptosis proteins, while the activity of the anti-apoptotic protein Bcl-2 is inhibited, leading to mitochondrial membrane instability and the release of apoptosis-related factors. These factors lead to caspase cascade hydrolysis, chromatin condensation and DNA fragmentation, finally leading to cell death [24, 25]. HSF1 is reported to be involved in the regulation of apoptosis in pancreatic cancer cells, although the specific mechanism remains to be elucidated [12, 13]. In the present study, we showed that Bcl-2 expression is downregulated following HSF1 silencing, while the expression of BAD, SMAC, cytochrome C, Apaf1, cleaved caspase 3, cleaved caspase 3 and cleaved caspase 9 is upregulated. Therefore, we speculate that HSF1 regulates the SMAC-mediated mitochondrial apoptotic pathway.

SMAC is a key regulator of the mitochondrial apoptotic pathway, which exists in mitochondria and activates caspase proteins by blocking apoptosis-inhibiting proteins (IAPs) [26]. The SMAC-mediated mitochondrial apoptotic pathway plays a key regulatory role in the development and progression of malignancies such as breast cancer [27], prostate cancer [28], and liver cancer [29]. Our study showed that downregulation of HSF1 promotes both SMAC expression and apoptosis in pancreatic cancer cells. Moreover, after SMAC silencing, XIAP protein expression was upregulated, while the expression of cleaved caspase 3 and cleaved caspase 9 proteins was downregulated. Furthermore, SMAC silencing resulted in decreased pancreatic cancer cell apoptosis, and significantly enhanced proliferation. HSF1 silencing reversed these effects. Furthermore, immunohistochemical analysis showed that SMAC expression was negatively correlated with HSF1 expression. Thus, our findings indicate that HSF1 inhibits the mitochondrial apoptosis pathway by regulating the expression of SMAC to promote pancreatic cancer cell proliferation and tumor growth.

HSPs are known to regulate mitochondrial apoptosis. HSP90 family proteins interact with cyclophilin D to inhibit mitochondrial permeability transition pore protein expression, protect mitochondrial membrane integrity, inhibit cell apoptosis and promote tumor cell growth [30]. HSP90 specific inhibitors can be used to regulate the mitochondrial apoptosis pathway leading to melanoma cell apoptosis, which inhibits tumor cell growth and proliferation [31]. HSF1 also regulates HSP70 to inhibit spermatogenic cell-dependent mitochondrial and death receptor pathway apoptosis [32]. Our results showed that the expression of HSP90, HSP60 and HSP27 proteins decreased after HSF1 silencing, and was associated with SMAC-mediated mitochondrial apoptosis. Therefore, we propose a new molecular model in which HSF1 regulates SMAC-mediated mitochondrial apoptosis by modulating the expression of HSPs, leading to tumorigenesis and development of pancreatic cancer.

## Conclusions

Our study shows that HSF1 is highly expressed in pancreatic cancer cells, and that its high expression is positively correlated with the degree of tumor differentiation and poor prognosis in pancreatic cancer patients. HSF1 expression was also found to be positively correlated with Ki-67 expression. Furthermore, our study confirms that HSF1 induces the expression of HSPs and regulates the SMAC-mediated mitochondrial apoptosis pathway to inhibit pancreatic cancer cell apoptosis and promote tumorigenesis. As far as we know, this study is the first report describing the role of HSF1 in the clinical pathology and prognosis of pancreatic cancer and demonstrating that HSF1 functions by regulating the SMAC-mediated mitochondrial pathway to inhibit pancreatic cancer cell apoptosis. Thus, our findings suggest that HSF1 may be an independent predictor of survival and prognosis in patients with pancreatic cancer and may serve as a target for the diagnosis and treatment of pancreatic cancer.

## Additional files

Additional file 1: Figure S1. Investigation of functional changes in PANC-1 cells following HSF1-siRNA # 2 transfection. A. PANC-1 cell proliferation was examined by CCK-8 assay. B. PANC-1 cell proliferation was evaluated by colony formation assay. C. Annexin V/PI double staining was used to detect PANC-1 cell apoptosis. D. PI staining analysis of the cell cycle of PANC-1 cells. (PDF 1337 kb)

Additional file 2: Figure S2. Low SMAC expression levels and negative association with the HSF1 expression in pancreatic cancer tissues. A. Representative image of immunohistochemical detection of SMAC protein expression in 50 pairs of pancreatic cancer and para-carcinoma tissue specimens. Scale bar, 50 μm. B. Semi-quantitative analysis of SMAC protein expression in pancreatic cancer and para-carcinoma tissues. Differences were analyzed by paired *t*-test and data represent the mean ± standard deviation of three independent experiments. ***P < 0.001. C. Correlation between HSF1 and SMAC detected in pancreatic cancer tissues by immunohistochemistry. Graph showing SMAC index in pancreatic cancer tissues with low or high expression of HSF1. (PDF 17136 kb)

## Abbreviations

HSF1: Heat shock factor 1
HSFs: Heat shock factors
HSPs: Heat shock proteins
OS: Overall survival
PI: Propidium iodide
RFS: Recurrence-free survival
ROC: Receiver operating characteristic
siRNA: Small interfering RNA
SMAC: Second mitochondria-derived activator of caspase
