# Supplementary figures and images for: Heat shock factor 1 inhibits the mitochondrial apoptosis pathway by regulating second mitochondria-derived activator of caspase to promote pancreatic tumorigenesis
Source: J Exp Clin Cancer Res. 2017 May 8;36:64. doi: 10.1186/s13046-017-0537-x (PMC5422968; doi:10.1186/s13046-017-0537-x)

**A**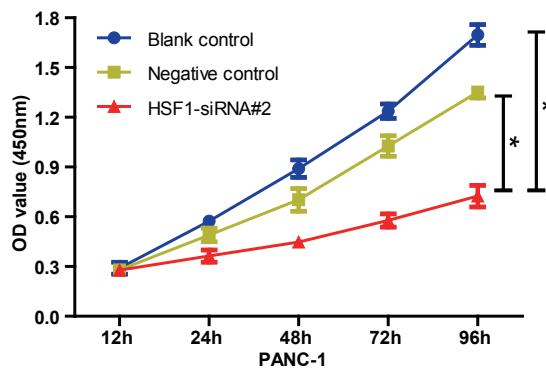**B**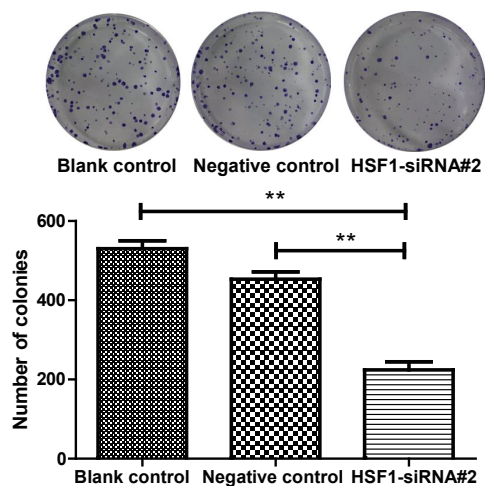**C**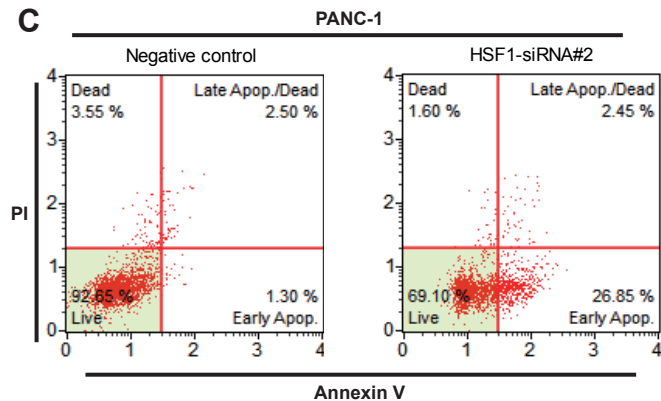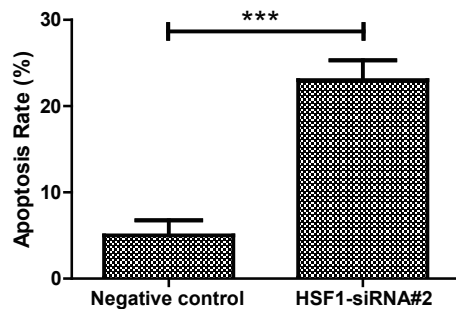**D**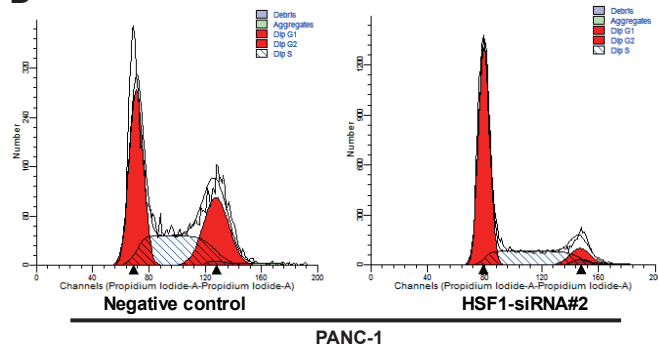

Supplement: Supplementary file 1 — Investigation of functional changes in PANC-1 cells following HSF1-siRNA # 2 transfection. A. PANC-1 cell proliferation was examined by CCK-8 assay. B. PANC-1 cell proliferation was evaluated by colony formation assay. C. Annexin V/PI double staining was used to detect PANC-1 cell apoptosis. D. PI staining analysis of the cell cycle of PANC-1 cells. (PDF 1337 kb) [file 13046_2017_537_MOESM1_ESM.pdf]

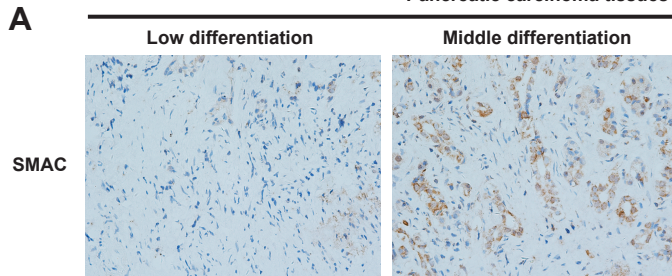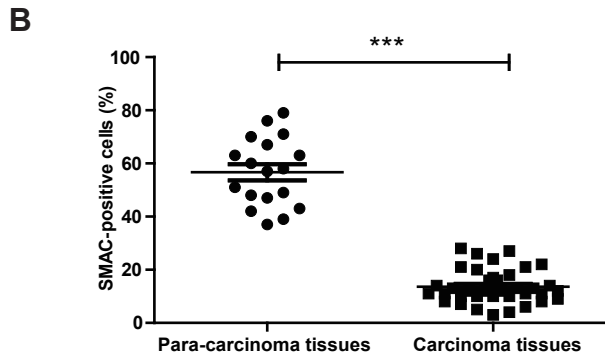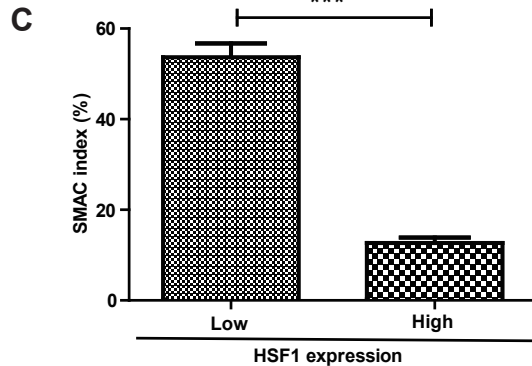

Supplement: Supplementary file 2 — Low SMAC expression levels and negative association with the HSF1 expression in pancreatic cancer tissues. A. Representative image of immunohistochemical detection of SMAC protein expression in 50 pairs of pancreatic cancer and para-carcinoma tissue specimens. Scale bar, 50 μm. B. Semi-quantitative analysis of SMAC protein expression in pancreatic cancer and para-carcinoma tissues. Differences were analyzed by paired t-test and data represent the mean ± standard deviation of three independent experiments. ***P < 0.001. C. Correlation between HSF1 and SMAC detected in pancreatic cancer tissues by immunohistochemistry. Graph showing SMAC index in pancreatic cancer tissues with low or high expression of HSF1. (PDF 17136 kb) [file 13046_2017_537_MOESM2_ESM.pdf]
